# Multidisciplinary reconstruction of complex cranio-scalp trauma in a patient with a domestic lion attack

**DOI:** 10.5339/qmj.2025.123

**Published:** 2025-12-15

**Authors:** Rand Y. Omari, Khalifa Al Alawi, Rabab Abdelrahman, Zaki Alyazji, Mahmoud Elsharkawy, Atalla Hammouda

**Affiliations:** 1 Plastic and Reconstructive Surgery Department, Hamad Medical Corporation, Doha, Qatar; 2 Trauma Intensive Care Unit and Department, Hamad Medical Corporation, Doha, Qatar *Email: romari@hamad.qa

**Keywords:** Qatar, large feline pet, lion attacks, degloving injury, scalp degloving, plastic surgery

## Abstract

**Introduction:**

Severe animal attacks by large predators, such as lions, are rare but devastating, often resulting in life-threatening injuries. These cases require immediate surgical intervention because of the complexity and severity of the injury. This report describes the multidisciplinary management of an adolescent who sustained extensive trauma following a pet-lion attack.

**Case Presentation:**

A 17-year-old male was brought to the emergency department by his father after a pet lion attack. He sustained severe injuries to the scalp, chest, arms, and face, along with a depressed skull fracture and pneumocephalus. He underwent emergent surgery involving dural repair with autologous temporalis fascia and cranial reconstruction using titanium mesh by the neurosurgery team, alongside extensive soft tissue debridement and layered closure by plastic surgeons. Postoperative care included broad-spectrum antibiotics, tetanus and rabies prophylaxis, and early physical therapy. The patient remained hospitalized for 5 days, during which he showed steady improvement and was discharged with preserved neurological function and well-healed surgical sites.

**Discussion:**

Animal attacks pose unique challenges in trauma care due to the complexity of injuries, high risk of infection, and the need for extensive reconstructive procedures. This case highlights the importance of a collaborative, multidisciplinary approach for managing such injuries and achieving optimal functional and aesthetic outcomes.

**Conclusion:**

Complex serious traumas in different locations of the body, especially craniofacial trauma. In this case, timely intervention and coordinated care resulted in a favorable outcome, with preserved neurological function and successful wound healing. This underscores the importance of rapid, comprehensive treatment and highlights the need for preventive measures to reduce the risk of such devastating injuries.

## 1. INTRODUCTION

The domestication of animals and the practice of keeping pets have been part of human society for thousands of years.^1^ Keeping a domesticated animal as a companion serves many roles, such as hunting, protection, companionship, and even therapeutic purposes. Pets commonly include animals, such as dogs, cats, birds, and other non-carnivorous mammals.^2^ However, a recent and concerning trend has emerged in which people are keeping carnivorous wild animals, such as tigers, lions, cheetahs, and mountain lions, as exotic pets.^3,4^ Attacks by large carnivores, though rare, often result in catastrophic outcomes. A recent global review of 5,089 large predator incidents reported a 32% fatality rate, with the majority of survivors sustaining significant trauma.^5^ Lions, in particular, are responsible for an estimated 250 human deaths annually worldwide, with many more nonfatal injuries underreported.^6^

These attacks typically involve deep bite wounds, avulsions, and fractures due to the animal’s bite force and claw strength. Prompt recognition of life-threatening injuries, early surgical management, and aggressive infection control are essential to prevent morbidity and mortality.

In this report, we present the case of a 17-year-old male who sustained multiple life-threatening injuries after being attacked by a lion. The case emphasizes the clinical decision-making, surgical complexity, and multidisciplinary coordination required in managing such trauma.

## 2. CASE PRESENTATION

A 17-year-old male with a known history of asthma was brought to the emergency department by his father 2 hours after a lion attack that occurred at a private residence. According to the patient’s father, the lion, 5 years old at the time and gifted to the patient by his father 3 weeks before the incident, was kept as an exotic pet but temporarily housed at a friend’s residence, as the friend already maintained other exotic animals and had established enclosures suitable for their care. This arrangement was to continue until the patient had completed preparations for his own facilities. The attack occurred during one of the patient’s visits to the friend’s residence.

**Figure 1:**
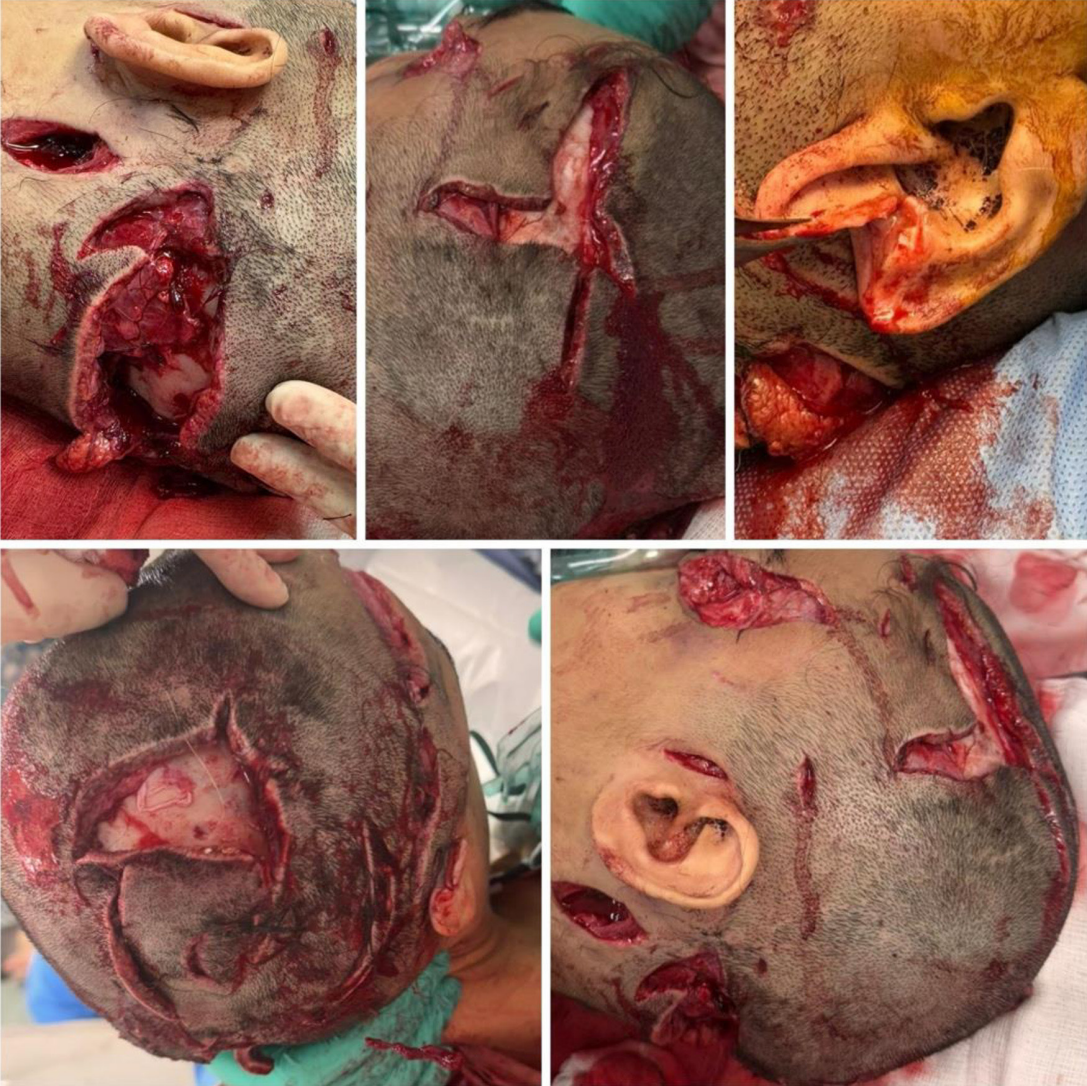
Severe scalp and left orbital degloving injuries were found on the patient during the primary survey in the emergency department following the lion attack.

Upon arrival, the patient had a Glasgow Coma Score of 15/15, was hypotensive (98/60 mmHg) and tachycardic (120 beats per minute), with signs of hemorrhagic shock. Primary survey revealed a circumferential degloving of the scalp with extensive bleeding, a large laceration involving the left lateral eye and severed ocular muscles, and multiple deep bite wounds over the chest and both upper limbs. The right auricle had a full-thickness laceration with exposed cartilage (Figure 1).

A computed tomography scan of the head showed a compound, comminuted depressed fracture of the left temporal bone with underlying pneumocephalus. The patient was rapidly stabilized and transferred to the operating room for emergent surgical intervention involving neurosurgery and plastic surgery teams.

**Figure 2:**
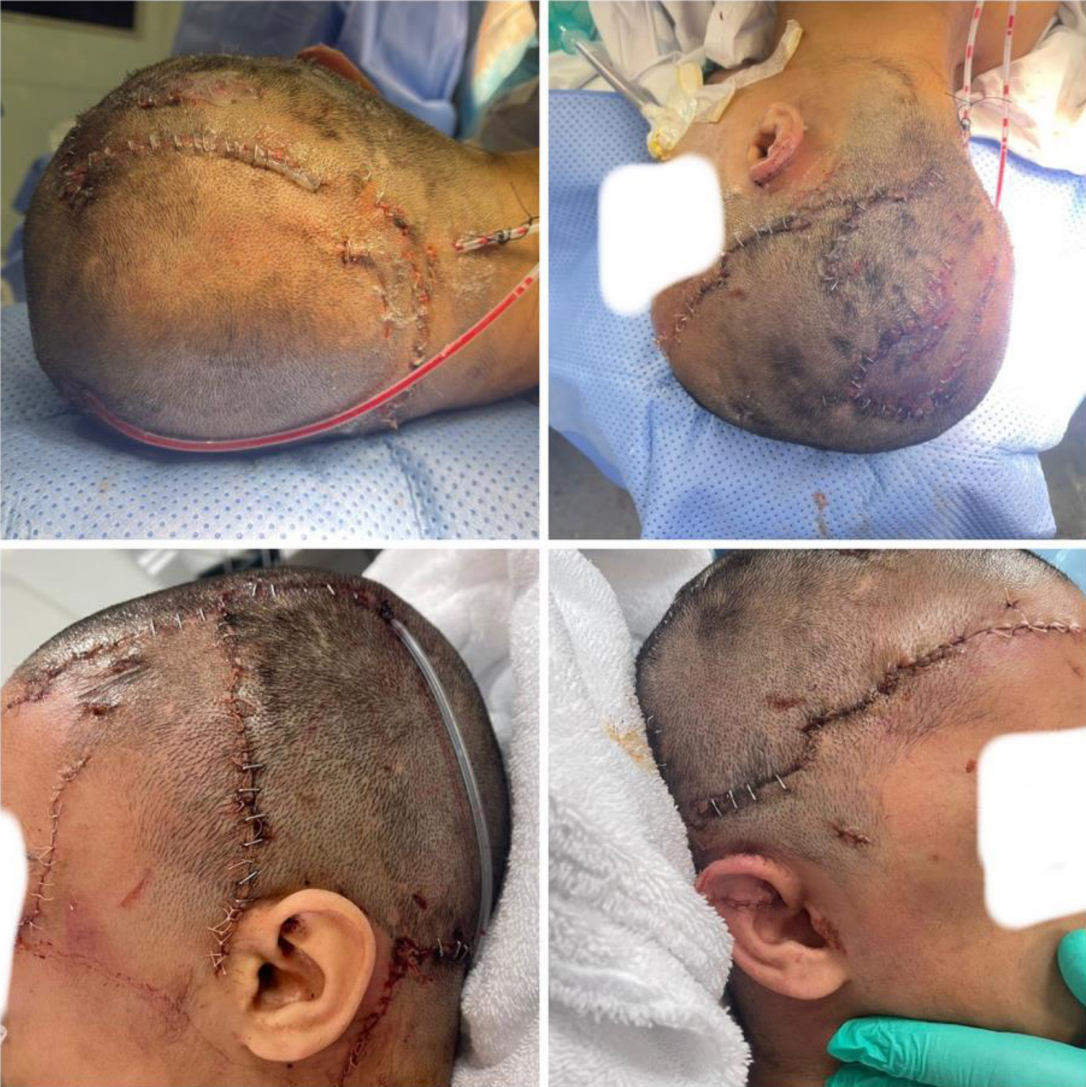
Immediate postoperative pictures of the patient following reconstructive scalp surgery.

Intraoperatively, large segments of the scalp showed full-thickness avulsion with areas of vascular compromise. The neurosurgical team debrided devitalized bones and identified a dural laceration beneath the fracture. Temporalis fascia was harvested and used for dural repair, followed by reconstruction of the skull defect with titanium mesh. The plastic surgery team then proceeded with meticulous debridement and irrigation of all bite wounds. The scalp was realigned and closed using layered, tension-free sutures to preserve perfusion. Vicryl 3-0 was used for subcutaneous approximation, Vicryl rapide 4-0 was used for more delicate areas, and staples were used for scalp skin-realignment (Figure 2). Two drains were inserted to minimize the formation of hematomas. Severed ocular muscles were reapproximated, and the external ear was repaired with cartilage realignment. Multiple deep wounds over the chest and arms were managed with irrigation, debridement, and closure.

**Figure 3:**
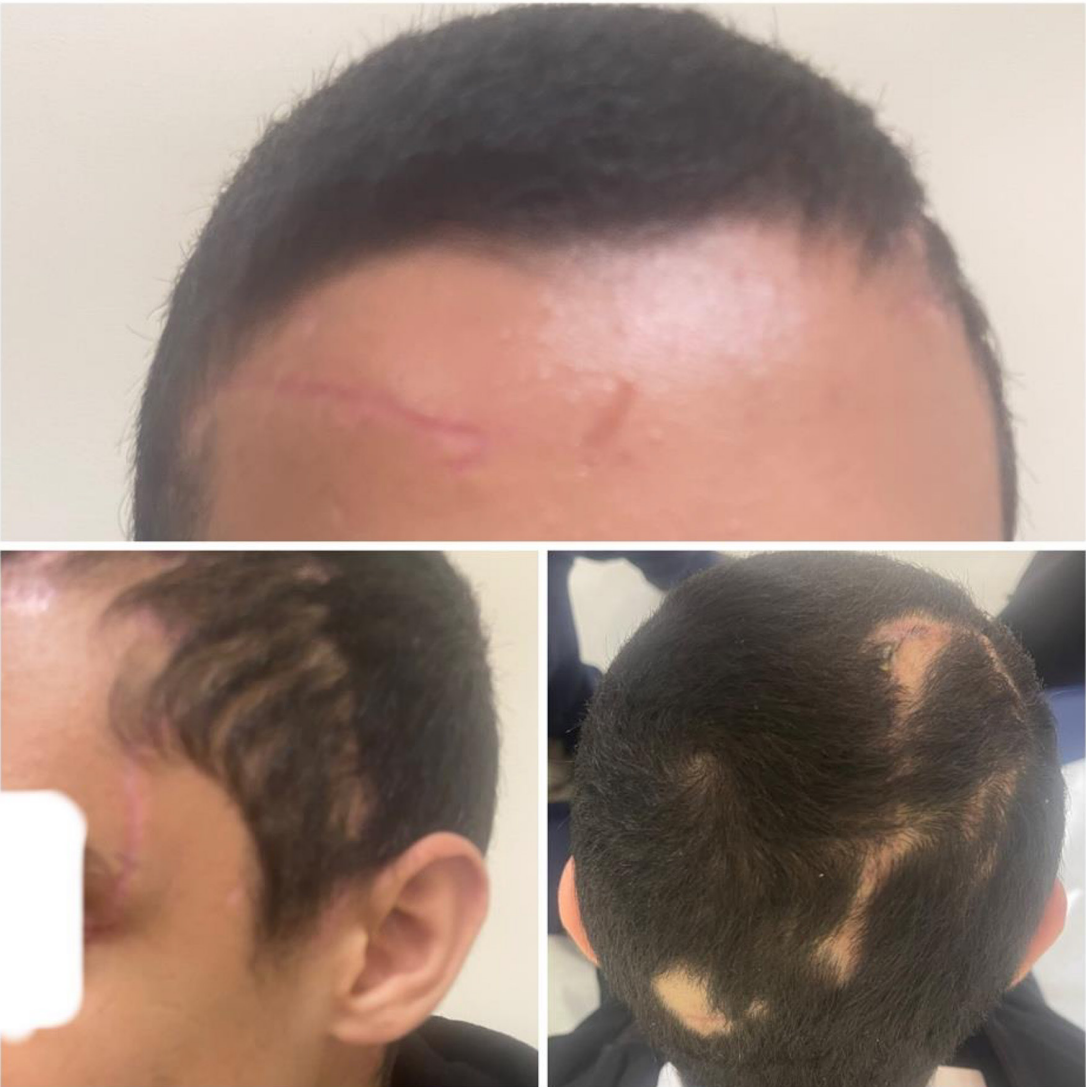
Pictures of the patient’s head 2 weeks post-discharge showing a well-perfused scalp, maintained hairline, and small scattered areas of alopecia.

Postoperatively, the patient was admitted to the Trauma Intensive Care Unit for 2 days, then was transferred to the Trauma Stepdown Unit. He received broad-spectrum antibiotics (piperacillin-tazobactam) and tetanus prophylaxis. Rabies post-exposure prophylaxis was initiated after consultation with infectious disease specialists. The wounds were inspected daily, drains were removed on postoperative day 5, and early physical therapy was initiated to preserve limb function. The patient was discharged on the 5th postoperative day. During the 2 weeks, all surgical sites had healed well, with viable scalp flaps, no areas of hair loss, and preserved neurological function (Figure 3).

## 3. DISCUSSION

Animal attacks, particularly those involving large predators, represent a unique challenge in trauma care. Lion-inflicted injuries are often severe due to their immense bite force, which can penetrate soft tissues, fracture bones, and cause significant bleeding.^6^ Head trauma, including concussions and skull fractures, is a common and serious consequence of such attacks. This case exemplifies the complexity of managing such trauma and highlights several critical aspects of care.

One of the primary challenges in this case was the management of the extensive scalp degloving injury. Scalp degloving injuries are rare but devastating, often associated with significant blood loss and the risk of ischemia to the scalp tissues.^7^ Early intervention with meticulous debridement, hemostasis, and tension-free closure is crucial to preserve the vascularity of the remaining scalp and minimize the risk of infection.

Additionally, the extensive bite wounds pose a significant risk of infection due to polymicrobial contamination from the lion’s oral flora.^8,9^ What is more, lion bites carry a high risk of bacterial infection due to deep tissue penetration and contamination with oral flora. Zoonotic pathogens like Pasteurella multocida and Capnocytophaga canimorsus increase the risk of secondary infections.^4,10^

The empirical use of broad-spectrum antibiotics is essential to prevent wound infections, cellulitis, and systemic sepsis.^11,12^ A study by Mowatt et al found that head and neck injuries are among the most fatal in large carnivore attacks.^13^ Immediate wound cleansing and prophylactic antibiotics are recommended for high-risk wounds. If infection develops, treatment should be guided by wound cultures. Early antibiotic intervention reduces complications and morbidity in Lion attack victims.^9^

Rabies and Tetanus prophylaxis are crucial due to documented cases of rabies-positive lions.^14^ World Health Organization (WHO) guidelines recommend wound care, rabies vaccine, and rabies immunoglobulin for post-exposure management.^15^ The Advisory Committee on Immunization Practices also advises tetanus vaccination and tetanus immunoglobulin for deep wounds.^16^

The surgical management of skull fractures with dural involvement requires precision to prevent complications such as meningitis, cerebrospinal fluid leaks, or long-term neurological deficits.^17^ Use of autologous fascia for dural repair and titanium mesh for reconstruction provides both functional and structural integrity.^18^

This case illustrates the critical importance of coordinated, multidisciplinary care in the management of complex animal bite trauma, particularly from exotic and large predatory animals such as lions. Effective treatment requires collaboration between trauma surgeons, neurosurgeons, plastic and reconstructive surgeons, infectious disease specialists, and critical care teams.^6^

Beyond the clinical scope, this case also highlights urgent public health concerns tied to the private ownership of exotic animals, predators like lions and tigers. Prevention must take precedence over treatment. Stronger regulatory oversight, public education, veterinary monitoring, and emergency preparedness are essential. While multidisciplinary care can address the aftermath, such incidents are largely preventable; this case reinforces the need for proactive policy, safety, and education to avoid avoidable harm.^4,19^

## 4. CONCLUSION

Severe injuries from lion attacks are rare but potentially devastating. In this case, prompt surgical intervention and coordinated multidisciplinary care enabled successful dural repair, cranial reconstruction, and soft tissue management, leading to a favorable recovery. The outcome highlights the importance of timely wound care, infection prevention, and rehabilitation. Moreover, this case underscores the urgent need for preventive public health measures, including stronger regulation of exotic animal ownership, education, and emergency preparedness to avoid such incidents.

## ETHICAL APPROVAL

Ethical approval was obtained from Hamad Medical Corporation, Abhath ID: MRC-04-25-828.

## CONSENT TO PARTICIPATE AND CONSENT FOR PUBLICATION

Written informed consent was obtained from the patient’s parents for the publication of this case and accompanying images. All authors were actively involved in the review, and all approved the final manuscript for publication.

## CONFLICT OF INTEREST

The authors of this review have no conflict of interest to declare.
